# Patient-specific implants are more stable than conventional miniplates in Le Fort I osteotomy in patients with clefts^✰^

**DOI:** 10.1016/j.jpra.2026.07.010

**Published:** 2026-07-07

**Authors:** Vilma Rouhiainen, Arja Heliövaara, Junnu Leikola, Juho Suojanen, Emma Juuri

**Affiliations:** a Cleft Palate and Craniofacial Center, Department of Plastic Surgery, Helsinki University Central Hospital and University of Helsinki, Helsinki, Finland; b Clinicum, Faculty of Medicine, University of Helsinki, Helsinki, Finland; c Päijät-Häme Joint Authority for Health and Wellbeing, Department of Oral and Maxillo-Facial Surgery, Lahti, Finland

**Keywords:** Patient-specific implants (PSI), Patient-specific osteosynthesis, Cleft lip and palate, Le Fort I, Orthognathic surgery

## Abstract

**Introduction:**

Virtual surgical planning (VSP) and patient-specific implants (PSI) have become standard in orthognathic surgery, demonstrating accuracy and stability comparable with miniplates.

**Aims:**

To compare postoperative skeletal stability of Le Fort I osteotomy using VSP with PSIs versus conventional cast model surgery with miniplate fixation in patients with clefts.

**Methods:**

Retrospective analysis of lateral cephalograms and medical records of patients who underwent Le Fort I osteotomy between 2017 and 2023 at the Cleft Palate and Craniofacial Center, Helsinki University Hospital. Horizontal and vertical surgical advancement and relapse were assessed by superimposing preoperative, immediate, and one-year postoperative cephalometric tracings. Statistical analyses included independent samples *t*-, Mann-Whitney *U*-, Chi-squared and Fischer’s exact test.

**Results:**

Fifty-nine non-growing patients with cleft lip and/or palate (29 males; median age 18.9 years) were categorized into two groups based on the fixation method (19 PSI, 40 miniplate). Mean maxillary advancement was greater with PSIs than miniplates (7.1 mm vs. 4.3 mm; p < 0.001). Mean vertical movement was -0.8 mm with PSIs vs. -3.8 mm with miniplates (p < 0.001), reflecting more impactions in the PSI group. Horizontal and vertical relapses were significantly smaller in the PSI group (horizontal: 0.28 mm vs. 0.9 mm, 4% vs. 21% of advancement; p < 0.001; vertical: 0.81 mm vs. 1.6 mm, 30% vs. 41% of movement; p = 0.004). Horizontal advancement correlated with relapse only with miniplates (r = 0.329, p = 0.038).

**Conclusions:**

PSI fixation in cleft Le Fort I osteotomy seems to provide greater horizontal and vertical stability than miniplates.

## Introduction

Patients with cleft lip and/or palate may develop maxillary hypoplasia caused by congenital dysmorphology, functional adaptations, and surgical iatrogenesis from previous primary and secondary cleft surgery.^1^ When a patient presents with midface deficiency, skeletal class III, and associated crossbites that lead to social, aesthetic, and functional problems, the discrepancy of the jaws can be corrected with orthognathic surgery.

Compared with the general population, a greater proportion of patients with clefts require orthognathic surgery (12.5–75%), with a greater likelihood in more severe cleft phenotypes, such as unilateral cleft lip and palate (UCLP) and bilateral cleft lip and palate (BCLP) .^2^ The reported prevalence of orthognathic surgery is highest in BCLP (38–71%)^3^, ^4^, ^5^ followed by UCLP (28–50%)^4^, ^5^, ^6^ and cleft palate only (CP) (13–18%).^6^^,^^7^ Some studies report lower overall prevalences (3–13%) across all cleft types.^8^

Compared with patients without clefts, achieving skeletal stability after Le Fort I osteotomy in patients with clefts is particularly challenging due to palatal scarring, maxillary bony defects, severe tooth agenesis, and the substantial advancements often required. Relapse rates vary broadly in patients with clefts (5–65% of advancement). Previous studies consistently show greater relapse rates in the vertical plane (mean 29%) than in the horizontal plane (mean 20%),^9^^,^^10^ with inferior repositioning of the maxilla relapsing more than impaction.^11^

Most of these studies pooled multiple cleft types in the same analysis. When considering cleft types individually, UCLP has a relapse rate of 18–25% in the horizontal dimension and 22–25% in the vertical dimension^12^, ^13^, ^14^, ^15^ BCLP shows variable results, both higher and lower relapse rates compared with UCLP.^11^^,^^15^^,^^16^ CP has the lowest horizontal relapse rate (9–16%), with vertical relapse comparable with other cleft types (17–30%).^16^^,^^17^

Three-dimensional virtual surgical planning (VSP) and patient-specific implants (PSI) have become universal in orthognathic surgery. Use of preoperative cone beam computed tomography (CBCT) to plan the surgery virtually with a 3D model, in contrast to traditional cast model surgery, is an accurate method for repositioning the maxilla.^18^^,^^19^ Individually manufactured osteosynthesis plates provide better accuracy and similar stability to stock plates.^20^, ^21^, ^22^, ^23^

Although the use of PSIs in patients with clefts has not yet been widely studied, early reports suggest that PSIs have promising accuracy and stability relative to stock plates.^24^^,^^25^

This study sought to compare the postoperative skeletal stability of Le Fort I osteotomy using VSP and PSIs with conventional cast model surgery with miniplate fixation in patients with clefts. We hypothesized that there is no difference between methods.

## Materials

### Patients

A retrospective analysis of lateral cephalometric radiographs and medical records was conducted for patients with completed growth who underwent Le Fort I osteotomy between January 2017 and December 2023 at the Cleft Palate and Craniofacial Center, Department of Plastic Surgery, Helsinki University Hospital.

Patients who underwent simultaneous bilateral sagittal split osteotomy or patients with a syndromic diagnosis were excluded. Growth completion was assessed using individual growth charts. Patients were categorized into two groups based on the fixation method used (PSI or miniplate).

Pre- and postsurgical orthodontics were performed with standard fixed appliances. Intermaxillary elastics were used postoperatively. Postsurgical splints were not used routinely and were applied only when possible re-enforced upper dental arch stability was considered beneficial.

### Surgical methods

All patients in the PSI group were operated on by the same senior surgical team (J.L. and J.S.). The patients in the miniplate group were operated on by one senior surgeon (J.L. or A.S.). The allocation of patients to a fixation method was driven by the surgeon preference of the method. None of the osteotomies were planned segmental. If previous osteotomy plates were present at surgery, they were first removed.

**Fig. 1:**
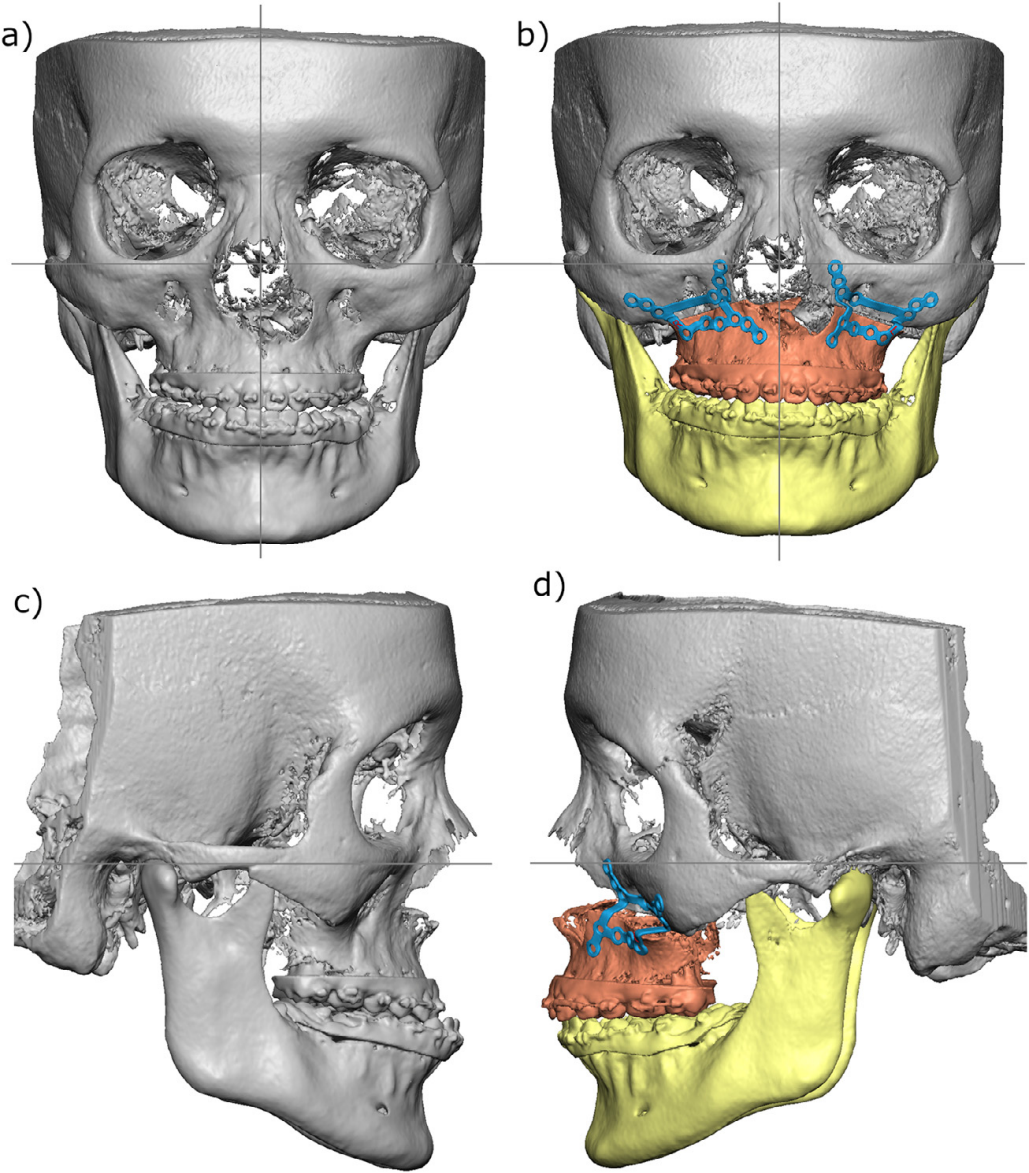
Frontal views of (a) preoperative and (b) 3D-planned postoperative models showing planned configuration of PSIs. Lateral (c) preoperative and (d) 3D-planned postoperative models. Mandible is marked with yellow, maxilla with orange, and PSIs with blue colour in the postoperative model.

**Fig. 2:**
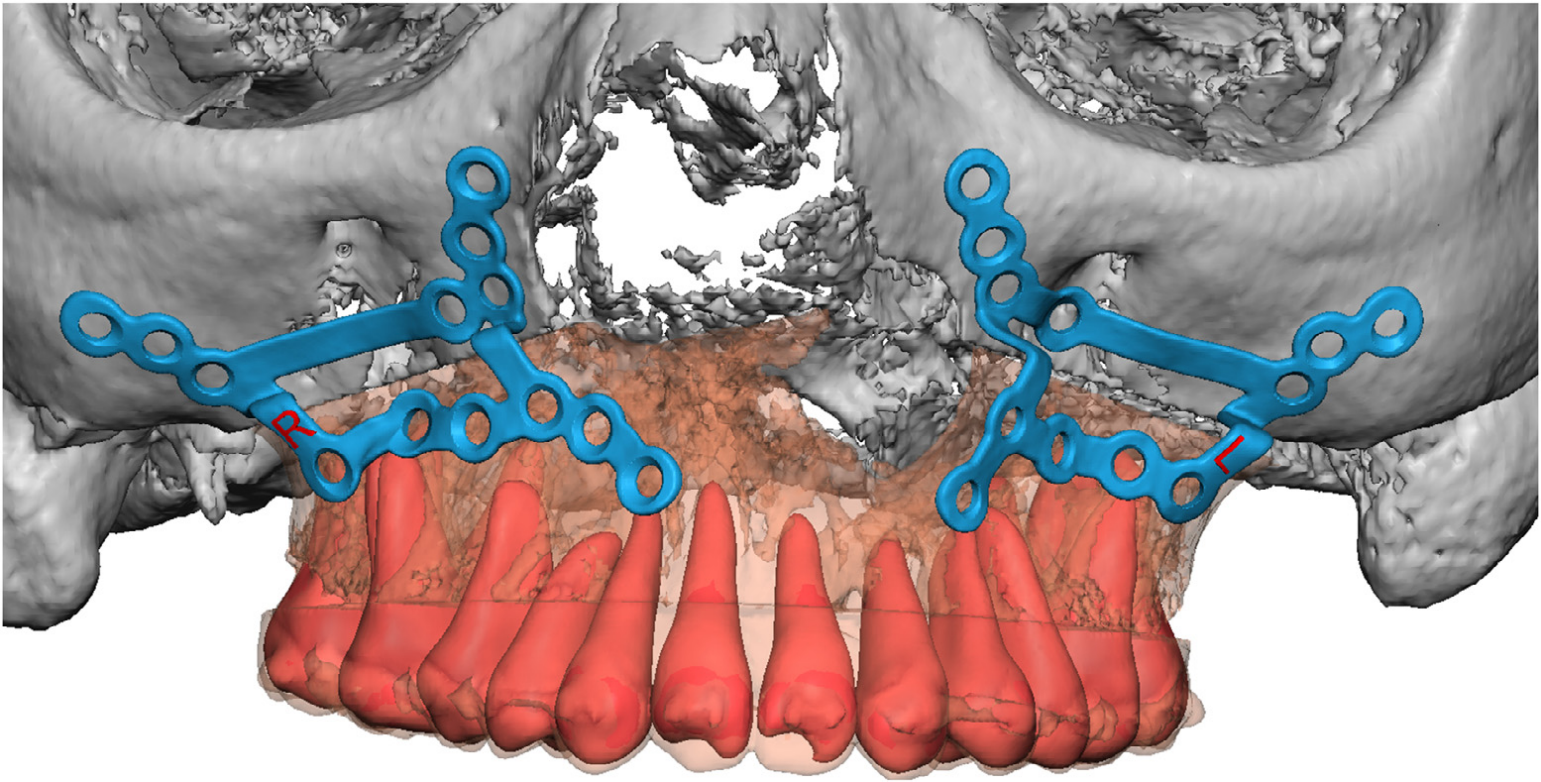
Frontal view of a patient-specific implant design example.

The standardized method of VSP and waferless Le Fort I osteotomy using surgical cutting and drilling guides and maxilla fixation with PSIs has been described in detail previously.^25^^,^^26^ A representative case is shown in Fig. 1, Fig. 2. PSIs were designed either as two separate plates or as a single midline-connected plate spanning both maxillary halves. PSIs were milled to thickness 0.8 mm and were fixed with KLS Martin maxDrive screws (Tuttingen, Germany) of diameter 2.0 mm or 2.3 mm. The exact number of fixation screws per patient was not systematically recorded in the medical records; however, PSI fixation typically involved 11–13 screws.

In the miniplate group, the fixation protocol was not fully standardized, and maxillary repositioning was most frequently achieved using occlusal wafers and maxillo-mandibular fixation. Osteosynthesis employed two plates on each side of the maxilla, either four L-plates or two Y- and two L-plates with 3 + 3 holes each. Comparable systems from three different manufacturers were used (MatrixORTHOGNATHIC/MatrixMIDFACE, DePuy Synthes, West Chester, PA, US; Arnett, KLS Martin, Tuttingen, Germany; Universal Orthognathic, Stryker, Portage, MI, US); plate thicknesses of 0.7 mm, 0.8 mm, and 0.6 mm were used, respectively, with the original screws provided for each system. Screw diameters varied from 1.7–2.3 mm. As in the PSI group, the exact number of fixation screws used per patient was not consistently documented and therefore could not be analysed.

### Cephalometric analysis

**Fig. 3:**
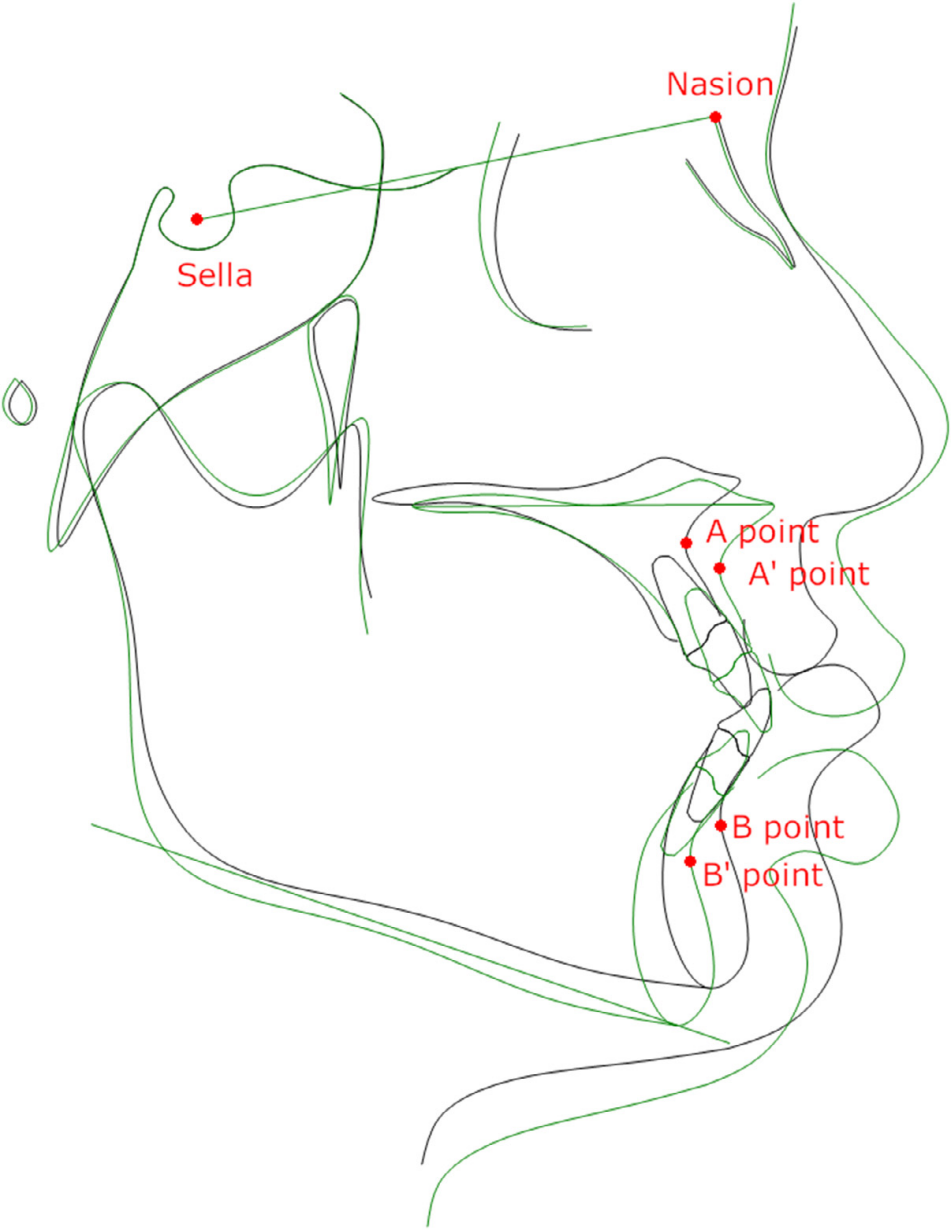
Superimposition of preoperative (black) and immediate postoperative (green) cephalometric tracings. Cephalometric points used for analysis are presented.

Lateral cephalograms at three different timepoints (preoperative, immediate postoperative, and one-year postoperative) were analysed. Surgical advancement and relapse in horizontal and vertical planes were quantified by superimposing preoperative, immediate, and one-year postoperative cephalometric tracings (Fig. 3).

For the PSI group, preoperative imaging was performed with CBCT/computed tomography (CT) with slice thickness 0.625–1 mm and gantry tilt 0° Lateral cephalograms were generated from CBCT/CT using orthogonal projection without magnification, a modality that has no significant cephalometric measurement differences when compared with conventional lateral cephalogram.^27^

Digital cephalometric analyses were conducted in Dolphin Imaging version 12.0.63 (Patterson Dental Holdings, Inc., Chatsworth, CA, US). Cephalograms produced from CBCT/CT were calibrated using the Dots-Per-Inch setting of the file. Lateral cephalograms were calibrated using the integrated ruler of the cephalostat. All analysis were performed by a single observer (V.R.).

Cephalometric tracings were oriented for superimposition using the Sella-Nasion line at Sella. Maxillary A point was used to determine the magnitude of surgical movement and postoperative relapse.

### Statistical methods

Chi-squared and Fischer’s exact test were used for comparing nominal variables. Independent samples *t*-test and Mann Whitney *U* were used for normally and non-normally distributed continuous variables, respectively. Distribution of the variables was confirmed with tests of normality and examining histograms. Pearson correlation coefficient (r) was used to compare covariances between continuous variables. Statistical analysis was performed with SPSS Statistics version 29.0.2.0 (IBM Corp., Armonk, NY, US).

## Results

### Patients

During the data collection period, altogether 72 patients underwent Le Fort I osteotomy, of which 13 were excluded due to missing radiographs or imaging obtained substantially outside the planned timepoints. Data from 59 patients (49% male) were analysed. Of these patients, 11 had a BCLP, 37 UCLP, eight CP, two cleft lip and alveolus (CLA) and one CP+CLA. Median age at surgery was 18.9 years (range 15.0–40.0). Primary cleft surgery was performed at the same centre in one or two stages during the first year of life, except for four patients who had their primary surgery abroad before relocating to Finland. All alveolar clefts were previously grafted with iliac crest bone. Two patients had previously undergone early Le Fort I osteotomy during growth for severe maxillary hypoplasia.

Patients were grouped by fixation method; 19 (32%) received PSIs and 40 (68%) miniplates. The groups were comparable by sex, age, cleft type, and presence of a pharyngeal flap but differed in ethnic distribution (Table 1).

**Table 1:** Patient characteristics.

	PSI (n = 19)	Miniplate (n = 40)	p-value
**Sex, n**			
Male	11 (57.9)	18 (45.0)	0.412
Female	8 (42.1)	22 (55.0)	
**Age at time of surgery, years**			
Median (IQR)	18.9 (3.8)	18.3 (3.9)	0.256
**Ethnic background, n**			
White	13 (68.4)	37 (92.5)	0.046⁎
Asian	5 (26.3)	2 (5.0)	
Hispanic	1 (5.3)	1 (2.5)	
**Cleft type, n**			
UCLP	11 (57.9)	26 (65.0)	0.857
BCLP	5 (26.3)	6 (15.0)	
CP	2 (10.5)	6 (15.0)	
CLA	1 (5.3)	2 (5.0)	
**Previous pharyngeal flap, n**			
Yes	4 (21.1)	4 (10.0)	0.416

IQR, interquartile range; PSI, patient-specific implant.

All values are n (%) unless otherwise indicated.

⁎ p < 0.05.

Operational characteristics and actual imaging intervals are shown in Table 2. Mean preoperative cephalometric angles SNA (Sella-Nasion-A point angle) and ANB (A point-Nasion-B point angle) illustrate the maxillary hypoplasia present in these patients, whereas SNB (Sella-Nasion-B point angle) represents the anteroposterior position of the mandible. Bone grafting was performed in nearly all osteotomy gaps; allograft alone was performed in 47 patients, autografts alone in three, and a combination in eight; only one patient did not receive a graft due to a small maxillary surgical advancement. Allograft materials used were Grafton® Putty (Osteo Tech, Eatontown, NJ, USA) or DBX® Putty (Musculoskeletal Transplant Foundation, Edison, NJ, USA).

**Table 2:** Operational characteristics.

	PSI (n = 19)	Miniplate (n = 40)	p-value
**Type of bone graft, n (%)**			
Allograft	18 (94.7)	29 (72.5)	0.221
Autograft	0 (0.0)	3 (7.5)	
Both	1 (5.3)	7 (17.5)	
None	0 (0.0)	1 (2.5)	
**Imaging intervals from operation, median (IQR)**			
Preoperative, days	50 (38)	63 (126)	0.961
Postoperative, days	2 (0)	2 (1)	0.071
Follow-up, months	13.2 (4.8)	12.4 (3.5)	0.209
**SNA, mean (SD)**			
Preoperative	74.7 (3.4)	76.0 (4.9)	0.297
Postoperative	81.4 (4.5)	80.3 (5.2)	0.427
Follow-up	81.2 (4.2)	79.4 (5.2)	0.196
**SNB, mean (SD)**			
Preoperative	80.3 (4.5)	78.7 (5.1)	0.243
Postoperative	79.2 (4.3)	77.4 (5.1)	0.204
Follow-up	80.5 (4.0)	77.8 (5.3)	0.054
**ANB, mean (SD)**			
Preoperative	−5.58 (3.1)	−2.66 (2.6)	<0.001⁎⁎
Postoperative	2.25 (2.9)	2.87 (2.2)	0.418
Follow-up	0.65 (2.9)	1.58 (2.4)	0.198

ANB, A point-Nasion-B-point; IQR, interquartile range; PSI, patient-specific implant; SD, standard deviation; SNA, Sella-Nasion-A-point; SNB, Sella-Nasion-B-point.

⁎⁎ p < 0.001.

### Surgical advancement

Mean horizontal maxillary advancement was greater with PSIs than miniplates (7.11 mm, SD 3.03 vs. 4.30 mm, SD 1.58; Table 3), with an overall mean advancement of 5.20 mm. Three patients in the PSI group exceeded 10 mm advancement.

**Table 3:** Mean magnitude of maxillary movements.

	PSI (n = 19)	Miniplate (n = 40)	p-value
**Surgical advancement, mm**			
Horizontal (SD)	7.11 (3.03)	4.30 (1.58)	<0.001⁎⁎
range	2.2–12.4	0.5–9.2	
Vertical (SD)	−0.79 (3.10)	−3.82 (2.27)	<0.001⁎⁎
range	−5.7–5.0	−8.8–0.6	
**Relapse, mm**			
Horizontal (SD)	0.28 (1.10)	0.90 (0.94)	0.031*
range	−1.9–2.1	−0.8–3.3	
% of advancement	4.0	21	
Vertical† (SD)	0.81 (0.69)	1.60 (1.34)	0.004*
range	0.1–2.5	0.1–4.8	
% of advancement	30	41	

In vertical surgical advancement, negative sign (-) means movement caudally.

In relapse negative sign (-) means movement to the same direction as surgical advancement.

PSI, patient-specific implant; SD, standard deviation.

⁎ p < 0.05.

⁎⁎ p < 0.001.

† calculated from absolute values.

Vertical movement of the cephalometric A point occurred both cranially and caudally, with 44 patients having clockwise and 15 counterclockwise rotation of the maxilla in the sagittal plane. The mean magnitude of vertical displacement was −0.79 mm (SD 3.10) in the PSI group and −3.82 mm (SD 2.27) in the miniplate group (negative indicates caudal movement). The absolute vertical movement, irrespective of direction, was 2.67 mm in the PSI group and 3.85 mm in the miniplate group (p = 0.044). The difference in magnitude of surgical movements was significant between the two groups in both horizontal and vertical axes (p < 0.001).

The preoperative ANB angle differed significantly between the groups, with the mean maxillary deficit being larger in the PSI group. The mean postoperative cephalometric angles reflected the improved skeletal relationships after surgery (Table 2), with no significant between-group differences, confirming that PSIs facilitated larger corrections.

### Relapse

Horizontal relapse differed significantly by fixation method. Mean relapse was 0.28 mm (range −1.9 to 2.1 mm, SD 1.10) with PSIs and 0.90 mm (range −0.8 to 3.3 mm, SD 0.94) with miniplates (p < 0.001; Table 3). Relative to group-specific mean advancements, this represented 4.0% and 21% relapse, respectively.

Vertical relapse occurred cranially and caudally; absolute values were used for analysing the amount of relapse. There was no significant difference in vertical relapses between clockwise- and counterclockwise-rotated maxillae (1 mm vs. 1.4 mm; p = 0.767), which confirmed the suitability of this conversion. In absolute values, the mean vertical relapse was 0.81 mm with PSIs and 1.60 mm with miniplates (p = 0.004), representing 30% and 41% of the absolute vertical movements, respectively.

In the miniplate group, greater horizontal advancement correlated with greater horizontal relapse (r = 0.329; p = 0.038). No significant correlation was observed in the PSI group (r = 0.404, p = 0.086), or when both groups were combined (r = 0.142, p = 0.283).

Additional analysis of relapse was performed for a subgroup with horizontal advancement ≥ 5 mm (14 PSI, 13 miniplate; mean advancements 8.46 mm, SD 2.22 and 5.92 mm, SD 1.17, respectively; p = 0.001). In this subgroup, mean relapse with PSIs was also significantly smaller than with miniplates (0.56 mm, SD 0.97 vs. 1.59 mm, SD 1.07; p = 0.015). Even in this subgroup, the mean advancement was significantly greater with PSIs, and the magnitude of advancement may have influenced the amount of relapse.

The reduction in mean SNA angle between immediate postoperative and one-year postoperative cephalometric angles seen in Table 2 reflects the already observed horizontal relapse. The mean reduction in SNA angle during follow up was significantly smaller with PSIs than with miniplates (−0.26°, SD 0.99 vs. −0.92°, SD 0.92; p = 0.015).

### Reliability

Intrarater reliability was assessed by re-digitizing 20 randomly selected radiographs after 6 months and calculating intraclass correlation coefficients (ICC) for the cephalometric points used for analysis.

ICC estimates were calculated with a single measurement, consistency, two-way mixed-effects model. ICCs indicated excellent reliability: point A, x-axis 0.993 (95% CI 0.983–0.997) and y-axis 0.980 (95% CI 0.950–0.992); point B, x-axis 0.989 (95% CI 0.971–0.995) and y-axis 0.970 (95% CI 0.926–0.988).^28^

## Discussion

Cleft-related palatal scarring, maxillary bony deficiencies, and need for substantial maxillary advancements pose significant challenges to the stability of Le Fort I osteotomy in patients with clefts. The aim of this study was to compare postoperative skeletal stability of Le Fort I osteotomy using VSP with PSIs versus conventional cast model surgery with miniplate fixation in patients with clefts.

We hypothesized that there would be no difference in stability between fixation methods. However, our results indicate superior horizontal and vertical stability with VSP and PSIs compared with conventional miniplate fixation, consistent with recent findings.^24^

The study cohort consisted predominantly of patients with UCLP or BCLP, even though CP has the largest incidence of cleft subtypes in Finland.^29^^,^^30^ The distribution of cohort cleft subtypes represents the higher demand of orthognathic surgery in patients with more severe clefts.^2^, ^3^, ^4^^,^ ^6^^,^^7^

Mean horizontal advancement was larger in the PSI group (7.1 mm) than in the miniplate group (4.3 mm), with overall advancement (5.2 mm) comparable with the upper range of prior series using stock plates in the same unit.^12^^,^^16^^,^^31^ Although Varidel et al. (2025) reported similar advancements with PSIs (median 6.3 mm), their miniplate group had larger advancements (median 7.0 mm) than our miniplate cohort. This discrepancy in the magnitude of surgical advancements between our study groups suggests a selection bias, i.e. patients with more substantial skeletal deficiencies were selected for the PSI group.

Horizontal relapses after Le Fort I osteotomy in patients with clefts have been reported at 20% of the advancement,^9^ with some studies reporting relapses up to 40%.^10^ Our miniplate group was consistent with these ranges, whereas the PSI group demonstrated a significantly lower relapse (4%) despite larger advancements. The range in relapses within the groups may be due to difference in cleft type, individual patient properties, bone quality or minor growth changes in adulthood, extent of scar tissue from prior repairs, and occlusal stability.

The clinical significance of the difference in relapse amount between methods can be debated, as the relapse was relatively small in both groups. Nevertheless, it is important to note that in some cases even small horizontal relapse of the maxilla can lead to unfavourable occlusion in the anterior region, such as anterior crossbite.

Inconsistent results have been reported on the correlation between magnitude of surgical advancement to the magnitude of relapse after Le Fort I osteotomy.^32^ In our study, the positive correlation between the magnitude of surgical advancement and the magnitude of relapse was confirmed in the miniplate group but not in the PSI group. Thus, in the overall data there was no correlation between these factors, likely due to the stabilizing effect of PSIs. It should also be noted that relapsing forces increase with the amount of the advancement; the miniplate group had significantly smaller advancements and it may be possible that the differences in the amount of relapse could be even more dramatic if the groups were matching.

The analysis of vertical stability is more complex as surgical movement of the maxilla in the vertical plane can be implemented cranially or caudally, depending on the surgical plan and initial relationship of the jaws. While subgrouping by the direction of vertical movement would be ideal, the sample sizes would become underpowered. However, using absolute values, PSIs showed improved vertical stability, consistent with Varidel et al.^24^

Bone grafting the osteotomy gap is essential to improve skeletal stability after large maxillary advancements^33^ and to reduce horizontal relapse.^34^ In this study, nearly all osteotomy gaps were grafted with autogenous bone, allogenous bone, or both. The heterogeneity of the grafting materials was due to differing preferences of the surgeons. Recent data indicate no significant differences in complications or reoperations between graft types in patients with clefts.^35^

In patients without clefts, PSIs achieve skeletal stability comparable with miniplates.^20^^,^^36^ Patients with clefts present with different anatomical challenges, including asymmetry and bone morphology. PSIs can provide an advantage by allowing accurate planning of the osteotomy line, plate and screw placement, and evaluation of bone thickness using the 3D model. PSIs are milled to precisely fit the contours of the maxillary bone, eliminating intraoperative bending, and PSIs provide broader surface coverage than miniplates. These features may contribute to improved stability. However, patient-specific implants are associated with substantially higher costs than conventional miniplates due to CAD/CAM design, virtual surgical planning, and custom manufacturing requirements.

The strengths of this study include the large sample of consecutive patients, which were all treated at one national centre. All patients were treated with the same standardized primary cleft protocol (except for four patients) and operations were performed by three surgeons.

Several limitations should be considered about the current study design. The retrospective design led to unequal group sizes due to the more recent adoption of PSIs. Surgeon preference influenced fixation method and variability in miniplate fixation implementation and the bone graft material used. In addition, the exact number of fixation screws used per patient was not available, which may possibly add variability to the results. Although the number of surgeons was small considering a sample of this size, the contribution of surgeons was not equal between the fixation methods, which can lead to operator-based bias. In addition, the amount of surgical advancement differed between groups, and analyses were based on two-dimensional cephalometrics, without accounting for rotational movements often used for midline correction. Such rotational movements could be accounted for with CBCT/CT imaging, which postoperatively have only recently become routine in our unit, enabled by reductions in radiation dose. Using two different imaging modalities (2D and 3D) for cephalometric analysis might also influence the accuracy of the measurements, even though ICC was considered excellent. Finally, the COVID-19 pandemic affected the imaging schedules, leading to exclusions; follow-up imaging from 6 months onward was accepted based on evidence that most relapse occurs within 6 months after surgery.^12^

To our knowledge, only one prior study has compared skeletal stability of Le Fort I osteotomy with PSIs vs. miniplates in patients with cleft lip and/or palate. Conducted at a national cleft centre, our study provides important additional evidence to the field of orthognathic surgery for this patient population. This study not only strengthens the existing evidence base but also reinforces the validity of prior reports on the implementation of newer technologies for Le Fort I osteotomy in cleft patients, an area where scientific evidence still is limited.^37^

Our data indicate that PSIs provide a stable fixation method in Le Fort I osteotomy in patients with clefts, facilitating larger advancements with minimal relapse. This suggests that more stable outcomes after cleft orthognathic surgery might be achievable even when substantial maxillary advancements are required.

## Conclusions

In this study, VSP with PSI fixation appears to result in less horizontal and vertical relapse after Le Fort I osteotomy than miniplates in patients with clefts. Further research should evaluate complications, operational efficiency, cost-effectiveness, and reoperation rates to determine the optimal fixation method for cleft Le Fort I osteotomy.

## Author contributions

Rouhiainen, V: Contributed to conception, design, data acquisition and interpretation, drafted and critically revised the manuscript.

Heliövaara, A: Contributed to conception, design, data acquisition and critically revised the manuscript.

Leikola, J: Contributed to conception, design, and critically revised the manuscript.

Suojanen, J: Contributed to conception, design, and critically revised the manuscript.

Juuri, E: Contributed to conception, design, data interpretation, drafted and critically revised the manuscript.

All authors gave their final approval and agreed to be accountable for all aspects of the work.

## Ethics

The research protocol was approved by Helsinki University Hospital (HUS/7851/2023) and principles outlined in the Declaration of Helsinki were followed. This study was performed following the STROBE guidelines.

## Funding

JS: Finnish Cultural Foundation and Ministry of Health research funds.

## Declaration of competing interest

The authors declared no potential conflicts of interest with respect to the research, authorship, and/or publication of this article.

## Data Availability

Data that support the findings of this study are available from the corresponding author upon reasonable request.
